# Assessment of body composition in breast cancer patients: concordance between transverse computed tomography analysis at the fourth thoracic and third lumbar vertebrae

**DOI:** 10.3389/fnut.2024.1366768

**Published:** 2024-04-23

**Authors:** Alex Daly, Lydia Newman, Alexandra Thomas, Alicia Munro, Cameron Spence, Joe Long, Jonathan Arnott, Kesta Durkin, David Layfield, Adam Heetun, Stephen Wootton, Ellen R. Copson, Ramsey I. Cutress

**Affiliations:** ^1^Cancer Sciences Academic Unit, Faculty of Medicine, University of Southampton, Southampton, United Kingdom; ^2^University Hospital Southampton NHS Foundation Trust, Southampton, United Kingdom; ^3^Salisbury District Hospital, Salisbury, United Kingdom; ^4^Human Development and Health, Faculty of Medicine, University of Southampton, Southampton, United Kingdom; ^5^NIHR Cancer and Nutrition Collaboration, Southampton, United Kingdom

**Keywords:** body composition, breast cancer, computed tomography, skeletal muscle, muscle attenuation, adiposity

## Abstract

**Introduction:**

Specific body composition markers derived from L3 axial computed tomography (CT) images predict clinical cancer outcomes, including chemotherapy toxicity and survival. However, this method is only applicable to those undergoing lumbar (L3) CT scanning, which is not universally conducted in early breast cancer cases. This study aimed to evaluate CT analysis at T4 as a feasible alternative marker of body composition in breast cancer.

**Method:**

All patients participated in the Investigating Outcomes from Breast Cancer: Correlating Genetic, Immunological, and Nutritional (BeGIN) Predictors observational cohort study (REC reference number: 14/EE/1297). Staging chest-abdomen-pelvic CT scan images from 24 women diagnosed with early breast cancer at University Hospital Southampton were analysed. Adipose tissue, skeletal muscle, and muscle attenuation were measured from the transverse CT slices’ cross-sectional area (CSA) at T4 and L3. Adipose tissue and skeletal muscle area measurements were adjusted for height. Spearman’s rank correlation coefficient analysis was used to determine concordance between body composition measurements using CT analysis at L3 and T4 regions.

**Results:**

Derived estimates for total adipose tissue, subcutaneous adipose tissue, and intramuscular adipose tissue mass following adjustment for height were highly concordant when determined from CSAs of CT slices at T4 and L3 (R_s_ = 0.821, *p* < 0.001; R_s_ = 0.816, *p* < 0.001; and R_s_ = 0.830, *p* < 0.001). In this cohort, visceral adipose tissue (VAT) and skeletal muscle estimates following height adjustment were less concordant when measured by CT at T4 and L3 (R_s_ = 0.477, *p* = 0.039 and R_s_ = 0.578, *p* = 0.003). The assessment of muscle attenuation was also highly concordant when measured by CT at T4 and L3 (R_s_ = 0.840, *p* < 0.001).

**Discussion:**

These results suggest that the CT analysis at T4 and L3 provides highly concordant markers for total adipose, subcutaneous adipose, and intramuscular adipose estimation, but not VAT, in this breast cancer population. High concordance between T4 and L3 was also found when assessing skeletal muscle attenuation. Lower concordance was observed for the estimates of skeletal muscle area, potentially explained by differences in the quantity and proportions of axial and appendicular muscle between the thorax and abdomen. Future studies will determine the value of T4 metrics as predictive tools for clinical outcomes in breast cancer.

## 1 Introduction

Obesity at the time of breast cancer diagnosis is associated with poorer outcomes, including shorter disease-free survival and overall survival. This aspect may be associated with increased rates of local or distant recurrence (1–4). Furthermore, obese patients with breast cancer are more likely to experience surgical and radiotherapy complications, chemotoxicity, and poorer treatment efficacy (1, 5, 6). Studies have also investigated the effects of physical exercise and other interventions related to body composition in breast cancer rehabilitation and survivorship (7). The mechanisms underlying the relationship between obesity and poorer outcomes are likely multifactorial. Proposed mechanisms include later diagnosis, more aggressive tumour types, and suboptimal chemotherapy treatment (8–11). Patients with cancer are at risk of developing sarcopenia and cancer cachexia, which is associated with a poorer prognosis in terms of recurrence, survival, and treatment toxicity (12). The processes underlying these changes in body composition are not fully understood but result from both cancer and treatment effects (13). A limitation of many studies that report cancer outcomes and body composition is that they only use body mass index (BMI), which is simply a ratio of weight over height squared and does not describe the individual components of body composition and so cannot account for variations in the proportions of fat, muscle, and bone (14). A small number of studies have, however, provided more detailed assessments of body composition by analysing cross-sectional areas (CSAs) of fat and muscle in the abdomen using computed tomography (CT), primarily at the third lumbar vertebrae (L3) level (15). Caan et al. (16) found that higher total adipose tissue and lower skeletal muscle quantities measured at L3 were associated with higher overall mortality rates in women with non-metastatic breast cancer. Similarly, Deluche et al. (17) reported that higher inter-muscular adipose tissue and lower skeletal muscle quantity measured by CT at L3 were associated with poorer disease-free survival in non-metastatic breast cancer. BMI was not associated with prognosis in these cohorts, emphasising the need for a more detailed body composition analysis (16, 17).

Although body composition/tissue analysis using CT at L3 may predict overall survival in patients with abdominal cancers who will routinely undergo abdominal imaging, UK guidelines and practice are that CT scans [which include the abdominal region (staging CT scans)] are only conducted in patients with breast cancer if there is a significant risk of metastasis (18–23). Consequently, there is a need for alternative methods of assessing body composition, as few patients with breast cancer routinely undergo CT scans of the abdominal (L3) region. CT analysis at the fourth thoracic vertebrae (T4) may provide an alternative method of assessing body composition in patients with breast cancer, as approximately 60% of patients with breast cancer undergo CT of the thoracic (T4) region for breast/chest wall radiotherapy planning purposes (24). The assessment of body composition parameters at T4 has been conducted in other cancers and was associated with clinical outcomes, including post-operative outcomes, respiratory function, and performance status (25, 26). The measurement of the pectoralis muscle area at T4 using CT was observed to be associated with worse disease-free and overall survival after adjustment for height in one cohort of patients with varied stages of breast cancer (27). Measurement of body composition parameters from a transverse slice at T4 is limited in breast cancer cohorts. We know only one prior published comparison between CT analysis of adiposity at T4 and L3 outside of breast cancer (26).

This study aimed to establish the feasibility of measuring body composition metrics from CT slices at T4 and assess whether body composition metrics measured at T4 are concordant with those measured at L3 in a population of women with early breast cancer.

## 2 Materials and methods

### 2.1 Study design and participants

**Figure 1:**
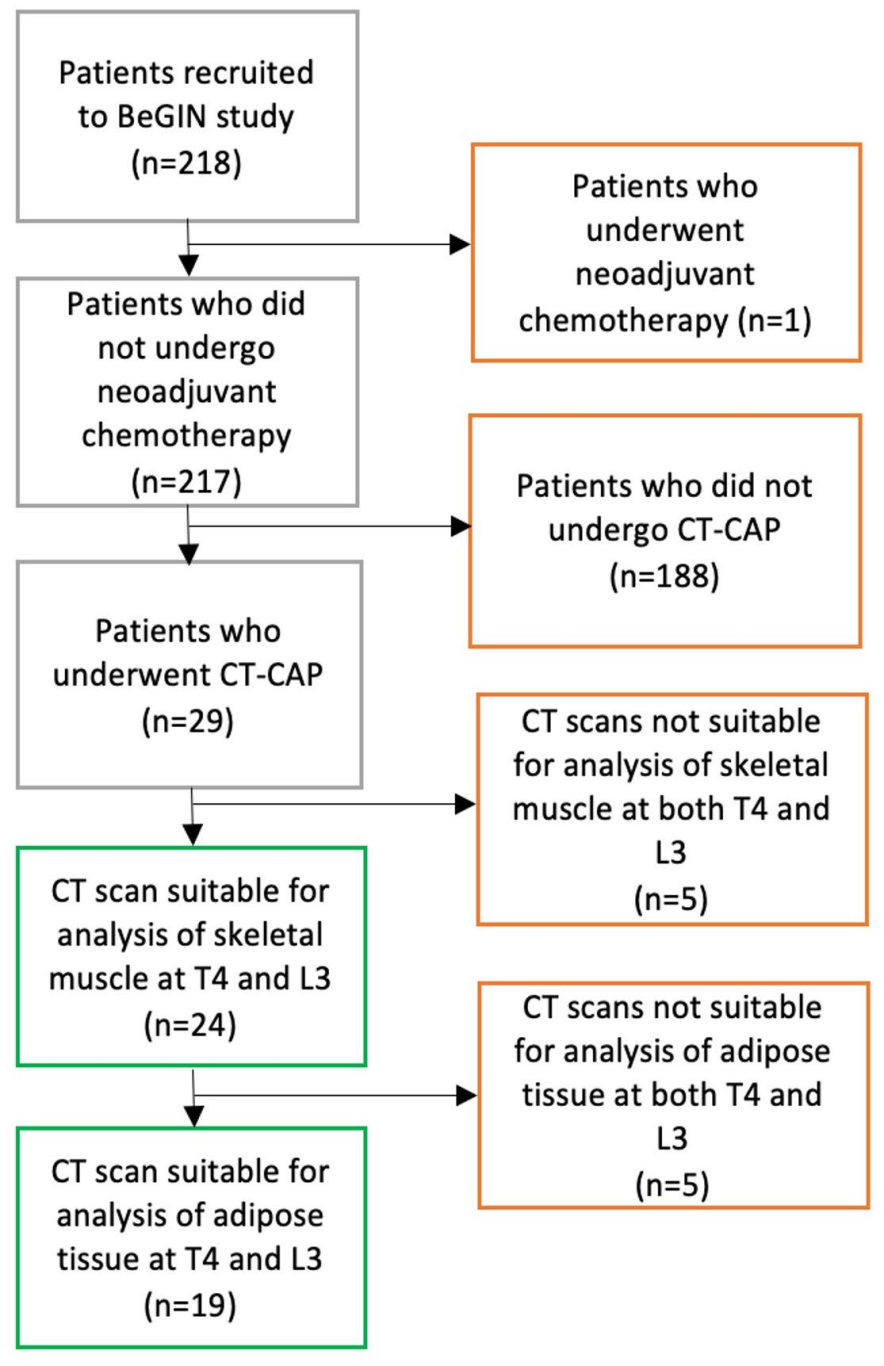
CONSORT diagram demonstrates the process of selecting participants for inclusion in study analysis. 218 patients were recruited to the BeGIN study; one patient was subsequently excluded because that patient underwent neo-adjuvant chemotherapy as per BeGIN study exclusion criteria. Among 217 patients who met the BeGIN study inclusion and exclusion criteria, 29 patients underwent a CT chest–abdomen–pelvis (CAP) as part of their routine breast cancer care. Of the 29 patients who underwent a CT CAP scan, 24 had CT scans suitable for the analysis of skeletal muscle at both T4 and L3, and 19 of those 24 patients had CT scans suitable for the analysis of adipose tissue at both T4 and L3.

The study participants were a subgroup of women diagnosed with early breast cancer who were recruited to a single-centre prospective observational cohort study at University Hospitals Southampton, “Investigating outcomes from breast cancer: Correlating genetic, immunological and nutritional predictors (BeGIN).” All participants gave written informed consent to use their anonymised data in this study. The research ethics committee approved the study (REC reference number: 14/EE/1297). Women were eligible for the BeGIN study if they were aged >18 years and diagnosed with invasive breast cancer or ductal carcinoma *in situ* after May 2015. Moreover, women were excluded if they underwent neoadjuvant chemotherapy or were aged <18 years. The participants included in this study were those patients from the BeGIN study cohort who had undergone routine diagnostic ‘staging’ CT chest-abdominal-pelvis whose CT images were suitable for analysis (see Figure 1 consort diagram). T4 or L3 CT images were deemed unsuitable for analysis if adipose tissue and skeletal muscle exceeded the image frame bilaterally. Linked anonymised patient information, including gender, age, smoking history, family history, tumour characteristics and histology, surgical management, chemotherapy, radiotherapy, biological, hormonal therapy, and chemotherapy toxicity as graded by the National Cancer Institute (NCI) Common Terminology Criteria for Adverse Events version 4.0, was extracted from the hospital electronic record system (28).

### 2.2 Body composition analysis using CT images

Routine CT scans were conducted pre- or post-operatively, depending on the patient’s clinical presentation and clinical indication. Two independent trained researchers conducted a body composition analysis. Anonymised CT slice images were selected by reference to body landmarks using *Radiant^™^ DICOM Viewer* software (29). The methodology for the body composition analysis at L3 was based on the Alberta protocol, and the most superior transverse CT slice at L3, where both transverse processes could be visualised, was selected (30). No protocols for the CT analysis of body composition at T4 had been published before the start of this study. Therefore, a novel methodology was developed with radiology specialist inputs. Based on radiology specialist advice, transverse CT slices at T4 were identified using bony landmarks (nine vertebrae superior from L1 and three vertebrae inferiorly from T1), then selecting the most superior image with a complete circular vertebral foramen present. T4 identification using these bony landmarks as reference points was more reliable than other landmarks, such as the aortic arch or carina location, which varied about the T4 vertebrae in our cohort when a pilot analysis was conducted.

**Figure 2:**
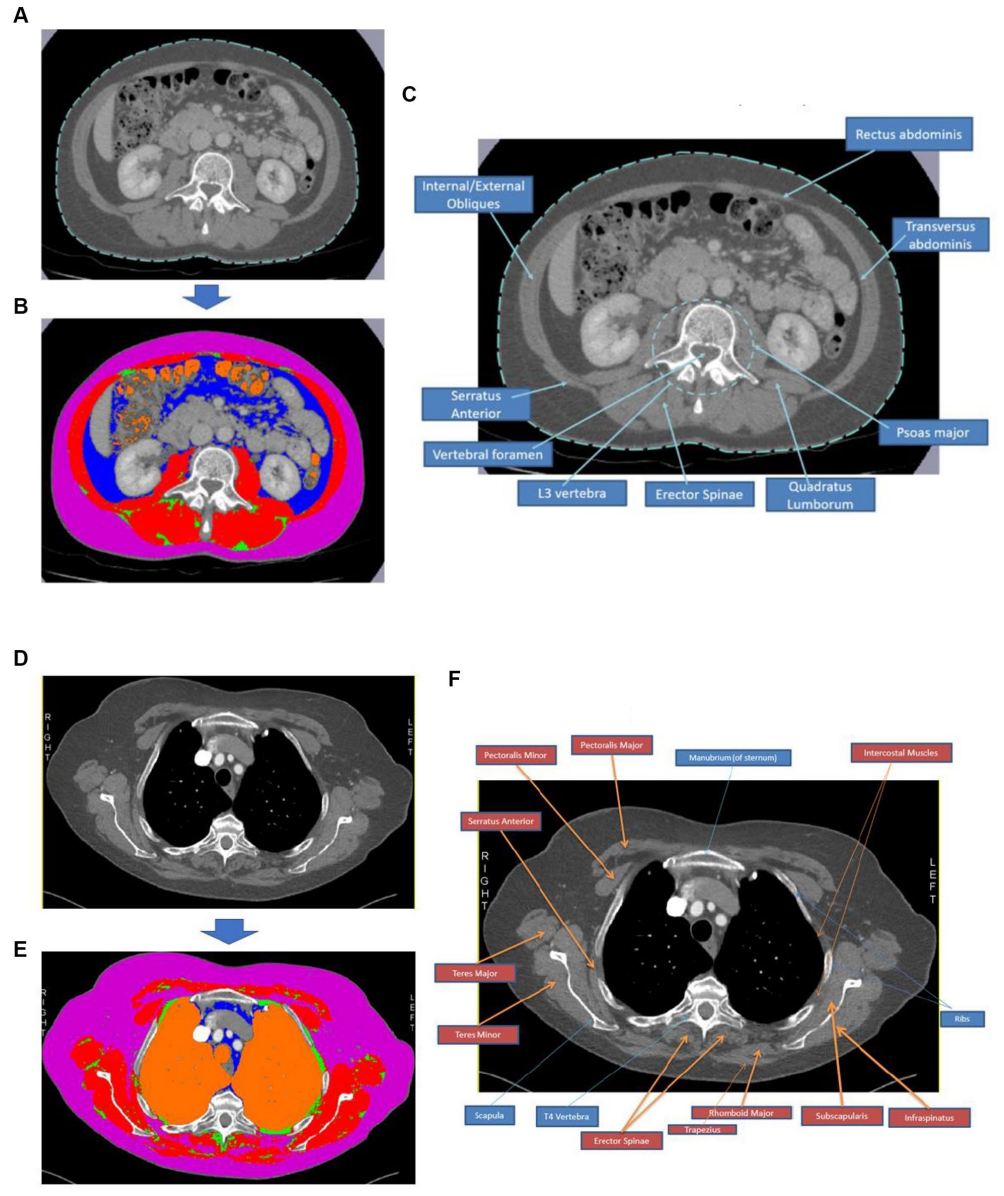
Body composition analysis process using CT images at T4 and L3. Image **(A)** shows an image of an L3 CT slice and **(B)** depicts the same slice following segmentation using *Sliceomatic^™^* software, where red = skeletal muscle, pink = subcutaneous adipose tissue, blue = visceral adipose tissue, green = intramuscular adipose tissue, and orange = air. Image **(C)** is an annotated CT image slice at L3 to demonstrate the critical muscle groups and bony landmarks typically visible in this region. Image **(D)** shows an image of a T4 CT slice, and **(E)** depicts the same slice following segmentation using *Sliceomatic^™^* software, where red = skeletal muscle, pink = subcutaneous adipose tissue, blue = visceral adipose tissue, green = intramuscular adipose tissue, and orange = air. Image **(F)** is an annotated CT image slice at T4 to demonstrate the key muscle groups and bony landmarks typically visible in this region.

Researchers manually selected segment regions of adipose and muscle from the T4 and L3 slice images using *Sliceomatic^™^* software (v5.0) to calculate CSAs and attenuations based on the Alberta protocol (30). Visceral adipose tissue (VAT), subcutaneous adipose tissue (SAT), intramuscular adipose tissue (IMAT), total adipose tissue (TAT = VAT + SAT + IMAT), and skeletal muscle were identified (see Figure 2). All muscle tissues visible at T4 were included. Where skeletal muscle or SAT were unilaterally incomplete in T4 images, the suitable image half was analysed, and the results per area were doubled accordingly. Complete protocols for image identification and analysis are shown in Supplementary material.

### 2.3 Statistical analysis

Statistical analysis was conducted using *SPSS^™^ Statistics v26.0.* Means were calculated for each data point from both researcher’s measurements, and the mean data were used in the final analysis. Index values were calculated for skeletal muscle and adipose tissues from the CSA measurements by dividing by height squared to adjust height (see Supplementary material). Values were normalised for height as previous research has demonstrated that whole-body skeletal muscle mass scales to a height approximately to the power of 2 (31). Adipose tissue scales less well to height with powers of approximately 2, but the same methodology was used for skeletal muscle and adipose tissues to allow comparison (32).

Paired *T*-tests were used where stated. Spearman’s rank correlation coefficients (R_s_) and regression coefficients (*R*^2^) were calculated to assess the degree of concordance between ranking variables at T4 and L3. Data are presented with x/y scatter plots with regression analysis and Bland–Altman plots to allow inter-researcher and inter-measure agreement comparison. Statistical significance was determined by *p*-values of <0.05.

## 3 Results

### 3.1 Patient characteristics and CT scan timing

Patient and tumour characteristics and breast cancer treatment received by the study cohort of 24 women reported are detailed in Tables 1–3. Study participants had a mean age of 61.3 years, ranging from 41 to 82 years. The majority (75%) of participants were classified as either ‘pre-obese’ (also often referred to as “overweight”; BMI 25–29.9 kg/m^2^) or ‘obese’ (BMI >30 kg/m^2^) as per World Health Organization criteria (33). All participants underwent surgical resection of their cancer, with more women undergoing a mastectomy (58.3%) compared to wide local excision (41.7%). Of women who also received chemotherapy (70.8%), a large proportion (64.7%) experienced at least one NCI grade 3 or higher chemotoxicity. As part of their routine treatment plans, participants also received radiotherapy (83.3%), endocrine (91.6%), and trastuzumab therapy (8.3%). CT scans were performed pre-operatively and post-operatively in 18 patients (75%) and 6 patients (25%), respectively. The timing of the scan ranged from 50 days pre-operatively to 122 days post-operatively, depending on the clinical indication.

**Table 1:** Demographic characteristics of the study cohort.

Demographic characteristics			Number of participants (*n*) (%)
Age (yrs)			
	18–39		0 (0.0%)
	40–49		5 (20.8%)
	50–59		6 (25.0%)
	60–69		8 (33.3%)
	70–79		4 (16.7%)
	>80		1 (4.2%)
BMI (mg/m2)			
	18.5–24.9		6 (25.0%)
	25.0–29.9		8 (33.3%)
	≥30		10 (41.7%)
Family history			
	Yes		5 (20.8%)
		Identified BRCA1/2 mutation	0 (0.0%)
		Family history (first- or second-degree relative)	5 (20.8%)
	None reported		18 (75.0%)
	Unknown		1 (4.2%)
	Total		**24**

This table describes the patient characteristics of the study cohort. BRCA = breast cancer gene.

**Table 2:** Tumour characteristics of the study cohort.

Tumour characteristics			Number of participants (*n*) (%)
Histological type			
	Invasive ductal		19 (79.2%)
	Lobular		2 (8.3%)
	Mixed		2 (8.3%)
	Papillary		1 (4.2%)
Grade			
	1		0 (0.0%)
	2		14 (58.3%)
	3		10 (41.7%)
Total tumour diameter			
	Mean (mm)		32.1
	Range (mm)		14.00–70.00
Axillary nodal involvement			
	Yes		17 (70.8%)
	No		7 (29.2%)
ER status			
	Positive		20 (83.3%)
	Negative		4 (16.7%)
HER2 status			
	Positive		4 (16.7%)
	Negative		20 (83.3%)
Total			**24**

This table describes the tumour characteristics of the study cohort. ER = oestrogen receptor, HER = human epidermal growth factor receptor.

**Table 3:** Treatment received by study cohort.

Treatment characteristics			Number of participants (*n*) (%)
**Surgical**			
Breast			
	Yes		24 (100%)
		Wide local excision	10 (41.7%)
		Mastectomy	14 (58.3%)
	None		0 (0.0%)
Axilla			
	Yes		22 (91.6%)
		Sentinel node biopsy	7 (29.2%)
		Axillary clearance	5 (20.8%)
		Both	10 (41.7%)
	No		2 (8.3%)
**Chemotherapy**			
Yes			17 (70.8%)
	Paclitaxel only		1 (4.2%)
	FEC-T		16 (66.7%)
No			7 (29.2%)
*Chemotoxicity*			
Not applicable			7 (29.2%)
Applicable			17 (70.8%)
Either no OR grade < 3 adverse event			6 (35.3%)
Grade ≥ 3 adverse event			11 (64.7%)
**Radiotherapy**			
Yes			20 (83.3%)
	Breast		10 (41.7%)
	Chest wall		10 (41.7%)
None			4 (16.7%)
**Endocrine therapy**			
Yes			22 (91.6%)
	Tamoxifen		7 (29.2%)
	Aromatase inhibitors		14 (58.3%)
	Consecutive		1 (4.2%)
None			2 (8.3%)
**Trastuzumab therapy**			
Yes			2 (8.3%)
No			22 (91.7%)
Total			**24**

This table describes the treatment received by the study cohort and chemotherapy toxicity. FEC-T = fluorouracil, epirubicin, cyclophosphamide, and docetaxel.

### 3.2 Inter-researcher agreement

**Figure 3:**
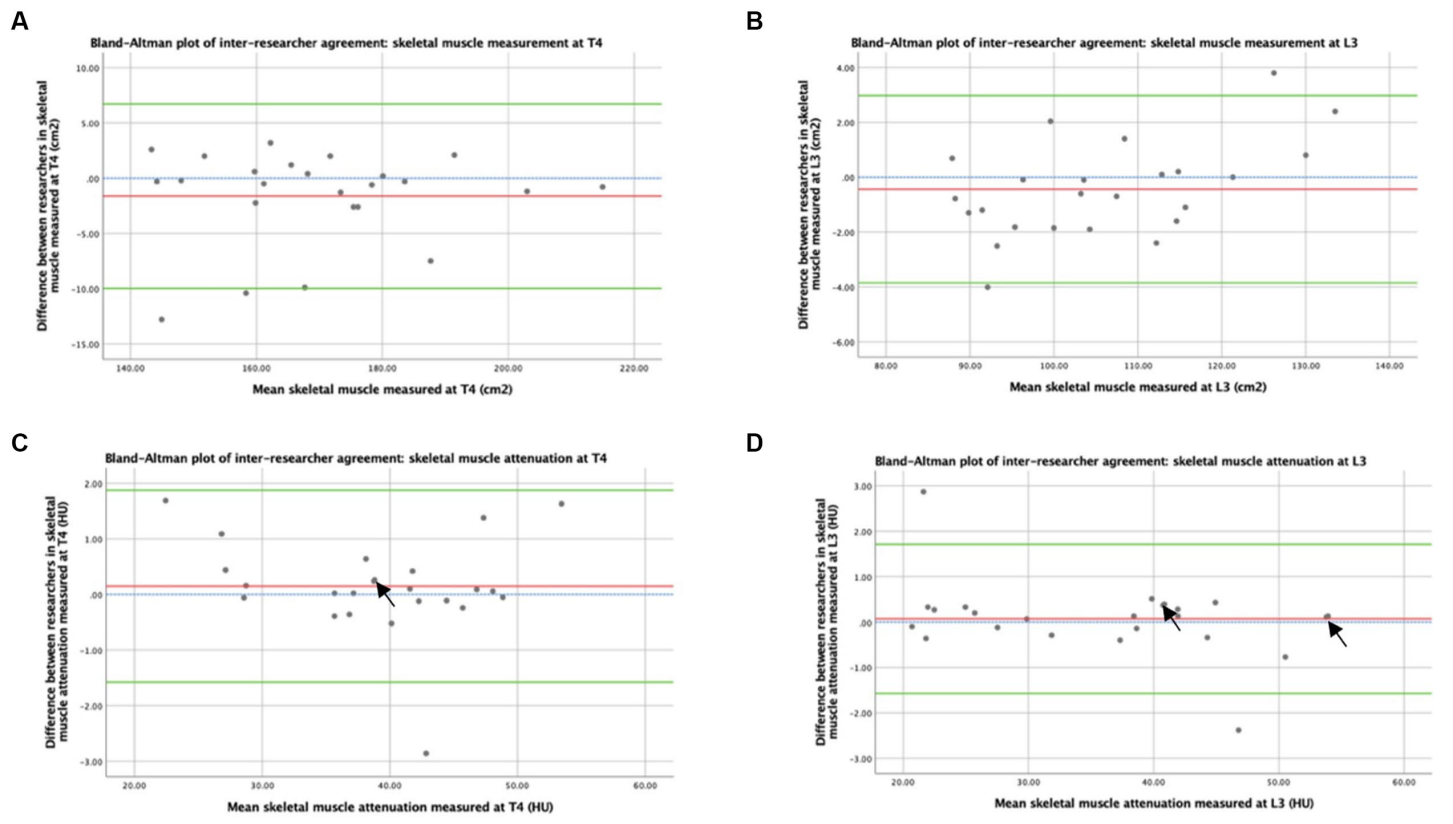
Concordance between researchers: Skeletal muscle was measured by CT. Graph **(A)** depicts a Bland–Altman plot of the average skeletal muscle cross-sectional areas (cm^2^) measured at T4 by the two researchers against the differences between these measurements (researcher 1 minus researcher 2). The mean difference ± standard deviation = −1.6 ± 4.3 cm^2^, where *n* = 24. Graph **(B)** depicts a Bland–Altman plot of the average skeletal muscle cross-sectional areas (cm^2^) measured at L3 by the two researchers against the differences between these measurements (researcher 1 minus researcher 2). The mean difference ± standard deviation = −0.4 ± 1.7 cm^2^, where *n* = 24. Graph **(C)** depicts a Bland–Altman plot of the average skeletal muscle attenuation (HU) measured at T4 by the two researchers against the differences between these measurements (researcher 1 minus researcher 2). The mean difference ± standard deviation = 0.15 ± 0.88 HU, where *n* = 24. Graph **(D)** depicts a Bland–Altman plot of the average skeletal muscle attenuation (HU) measured at L3 by the two researchers against the differences between these measurements (researcher 1 minus researcher 2). The mean difference ± standard deviation = 0.07 ± 0.84 HU, where *n* = 24. No difference (blue interrupted line), mean difference (red line), and 95% upper and lower limits of agreement (mean difference ± 1.96*standard deviation, green lines) are indicated. Arrows indicate two overlapping data points.

**Figure 4:**
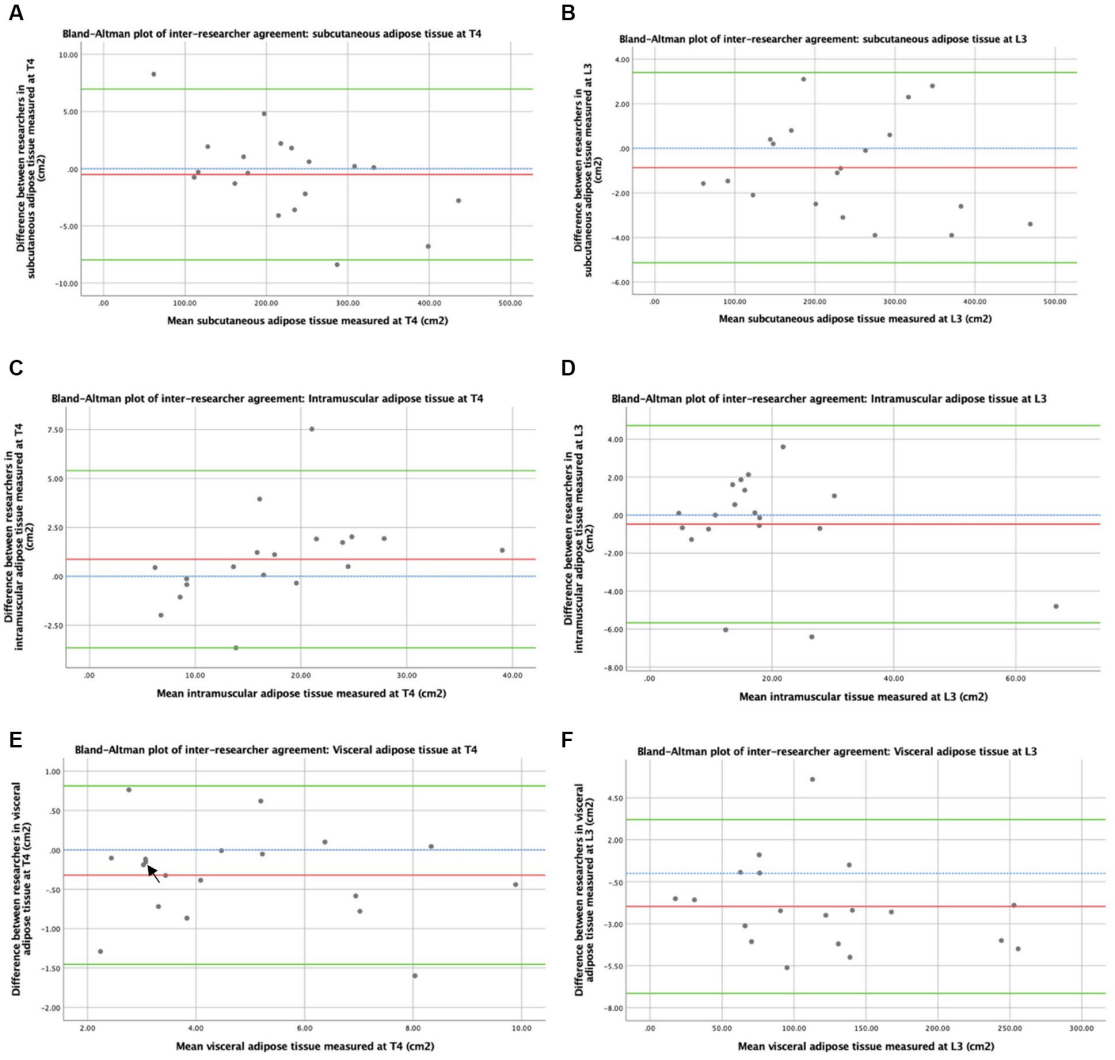
Concordance between researchers: adiposity measured by CT. Graph **(A)** depicts a Bland–Altman plot of the average subcutaneous adipose tissue cross-sectional areas (cm^2^) measured at T4 by the two researchers against the differences between these measurements (researcher 1 minus researcher 2). The mean difference ± standard deviation = −0.5 ± 3.8 cm^2^, where *n* = 19. Graph **(B)** depicts a Bland–Altman plot of the average subcutaneous adipose tissue cross-sectional areas (cm^2^) measured at L3 by the two researchers against the differences between these measurements (researcher 1 minus researcher 2). The mean difference ± standard deviation = −0.9 ± 2.2 cm^2^, where *n* = 19. Graph **(C)** depicts a Bland–Altman plot of the average intramuscular adipose tissue cross-sectional areas (cm^2^) measured at T4 by the two researchers against the differences between these measurements (researcher 1 minus researcher 2). The mean difference ± standard deviation = 0.9 ± 2.3 cm^2^, where *n* = 19. Graph **(D)** depicts a Bland–Altman plot of the average intramuscular adipose tissue cross-sectional areas (cm^2^) measured at L3 by the two researchers against the differences between these measurements (researcher 1 minus researcher 2). The mean difference ± standard deviation = −0.5 ± 2.7 cm^2^, where *n* = 19. Graph **(E)** depicts a Bland–Altman plot of the average visceral adipose tissue cross-sectional areas (cm^2^) measured at T4 by the two researchers against the differences between these measurements (researcher 1 minus researcher 2). The mean difference ± standard deviation = −0.3 ± 0.6 cm^2^, where *n* = 19. Graph **(F)** depicts a Bland–Altman plot of the average visceral adipose tissue cross-sectional areas (cm^2^) measured at L3 by the two researchers against the differences between these measurements (researcher 1 minus researcher 2). The mean difference ± standard deviation = −2.0 ± 2.6 cm^2^, where *n* = 19. No difference (blue interrupted line), mean difference (red line), and 95% upper and lower limits of agreement (mean difference ± 1.96*standard deviation, green lines) are indicated. Arrows indicate two overlapping data points.

Bland–Altman plots of the inter-researcher agreement for skeletal muscle and adipose tissue measurements at T4 and L3 are shown in Figures 3, 4, respectively. Generally, small differences were observed between researchers’ measurements, and no overt measurement biases were identified. The mean difference in skeletal muscle CSA between researchers was 1.6 cm^2^ at T4 and 0.4 cm^2^ at L3. A mean difference of 0.15HU was observed between research measurements of skeletal muscle attenuation at T4 and a difference of 0.07HU at L3. When researchers measured SAT, a 0.5cm^2^ mean difference was observed at T4 and 0.9cm^2^ at L3. A mean difference of 0.9cm^2^ was observed at T4 and 0.5cm^2^ at L3 in intramuscular adiposity measured between researchers. Mean differences of 0.3cm^2^ and 2.0cm^2^ were observed between researchers when VAT was measured at T4 and L3, respectively.

### 3.3 Adiposity assessment by CT at T4 and L3

**Figure 5:**
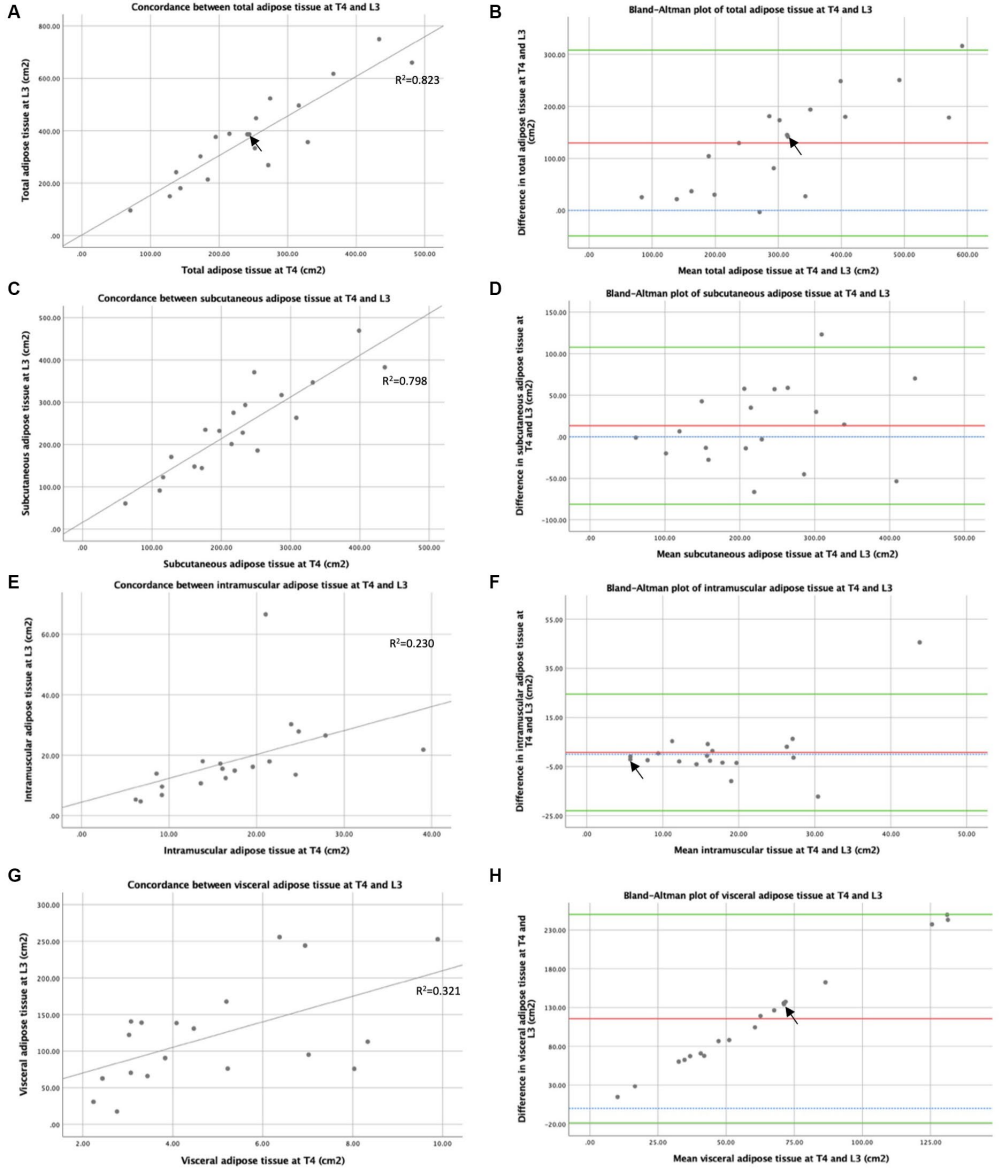
Concordance between T4 and L3: adipose tissue measured by CT. Concordance between CT at T4 and L3 of total adipose tissue cross-sectional areas (cm^2^) **(A)**, subcutaneous adipose tissue cross-sectional areas (cm^2^) **(C)**, intramuscular adipose tissue cross-sectional areas (cm^2^) **(E)**, and visceral adipose tissue cross-sectional areas (cm^2^) **(G)** are depicted by x-y scatter plots. Lines of best fit are shown, based on regression equation analysis, where *R*^2^ = 0.823 **(A)**, *R*^2^ = 0.798 **(C)**, *R*^2^ = 0.230 **(E)**, and *R*^2^ = 0.321 **(G)**. Graph **(B)** depicts a Bland–Altman plot of the averages between total adipose tissue cross-sectional areas (cm^2^) measured by CT at T4 and L3 against the differences between these measures (L3 minus T4). Mean difference ± standard deviation = 129.6 ± 91.2 cm^2^. Graph **(D)** depicts a Bland–Altman plot of the averages between subcutaneous adipose tissue cross-sectional areas (cm^2^) measured by CT at T4 and L3 against the differences between these measures (L3 minus T4). Mean difference ± standard deviation = 13.3 ± 48.3 cm^2^. Graph **(F)** depicts a Bland–Altman plot of the averages between intramuscular adipose tissue cross-sectional areas (cm^2^) measured by CT at T4 and L3 against the differences between these measures (L3 minus T4). Mean difference ± standard deviation = 0.8 ± 12.1 cm^2^. Graph **(H)** depicts a Bland–Altman plot of the averages between visceral adipose tissue cross-sectional areas (cm^2^) measured by CT at T4 and L3 against the differences between these measures (L3 minus T4). Mean difference ± standard deviation = 115.6 ± 68.5 cm^2^. In graphs **(B,D,F,H)**, no difference (blue interrupted line), mean difference (red line), and 95% upper and lower limits of agreement (mean difference ± 1.96*standard deviation, green lines) are indicated. For all graphs *n* = 19, arrows indicate two overlapping data points.

The concordance between TAT as CSAs measured by CT at T4 and L3 is shown in Figure 5A. A strong positive correlation between TAT following normalisation and height by CT CSA at T4 and L3 suggested that CT analysis at T4 and L3 ranked patients similarly by adiposity (R_s_ = 0.821, *p* < 0.001). When averages for TAT indexes were plotted against the differences between measurements at T4 and L3 in a Bland–Altman plot (see Figure 5B), a bias effect was observed whereby adiposity was greater at L3 than at T4. On average, total adiposity was 47.0cm^2^m^−2^ greater at L3 compared to T4, reflecting increased adiposity in the abdominal vs. the thoracic region. In addition, a more significant difference was observed between adiposity measured at L3 and T4 in individuals with higher mean adiposity. As shown in Figure 5C, similarly, strong concordance was observed between the SAT index measured at T4 and L3 (R_s_ = 0.816, *p* < 0.001). On average, SAT was 4.5cm^2^m^−2^ greater at L3 compared to T4. The correlation between the IMAT index measured at T4 and L3 (see Figure 5E) was similarly high to that observed with total and SAT (R_s_ = 0.830, *p* < 0.001). The Bland–Altman analysis reflected minor differences between IMAT when measured at T4 and L3 in most participants (see Figure 5F). Measurements at T4 were 0.2cm^2^m^−2^ lower on average than at L3. Spearman’s rank correlation coefficient demonstrated a weakly positive correlation between the visceral adiposity index estimated from T4 and L3 (R_s_ = 0.477, *p* = 0.039), suggesting that the CT analysis at T4 and L3 ranks patients less similarly than the other adipose tissue types measured (Figure 5G). On average, visceral adiposity was 42.3 cm^2^m^−2^ higher at L3 than at T4. The Bland–Altman analysis demonstrates a bias whereby, as mean visceral adiposity increases, a greater difference is observed between VAT measured at T4 and L3 (see Figure 5H).

### 3.4 Skeletal muscle assessment by CT at T4 and L3

**Figure 6:**
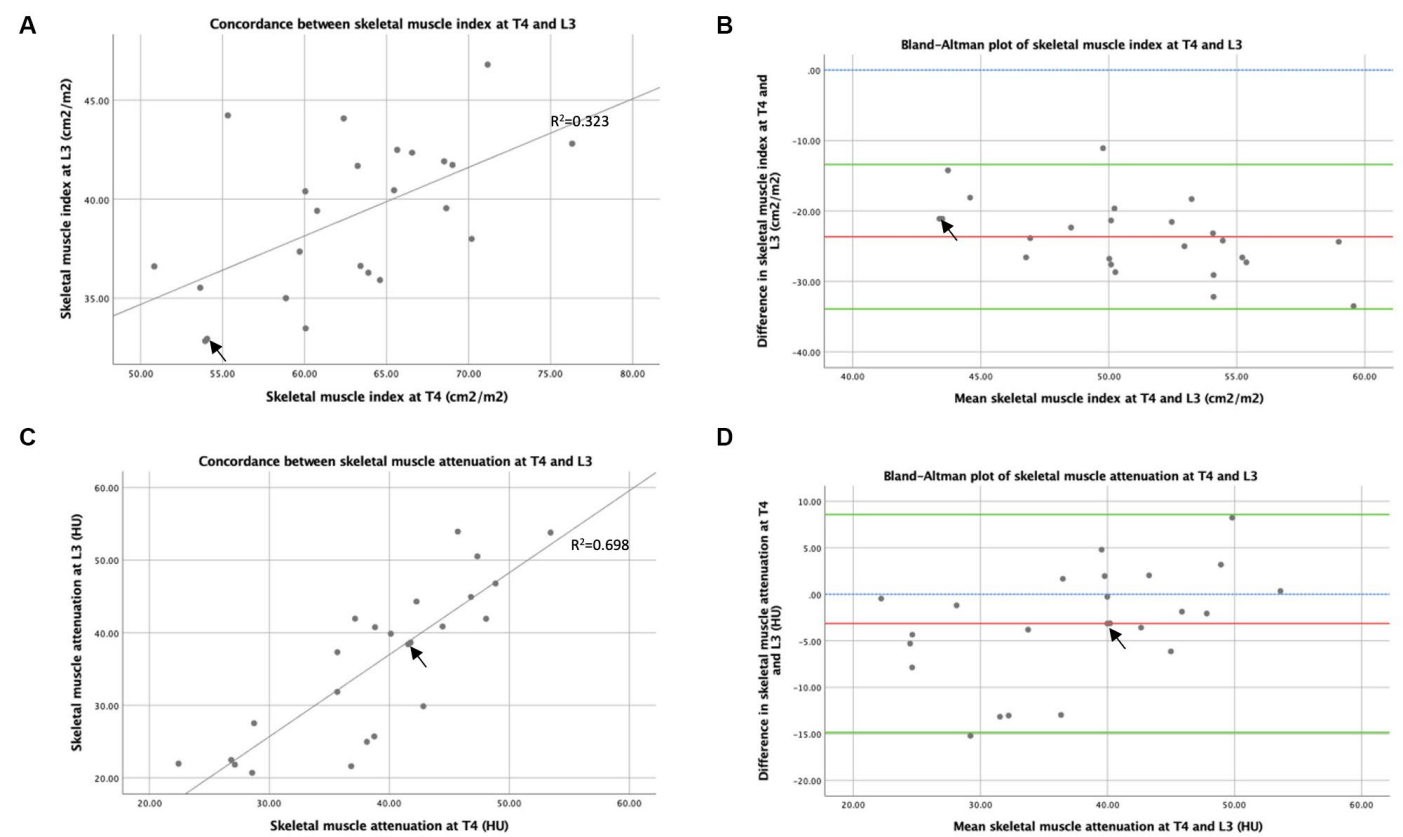
Concordance between T4 and L3: skeletal muscle area and attenuation measured by CT. Graph **(A)** is an x–y scatter plot of the concordance between skeletal muscle cross-sectional area measured by CT at T4 and L3 as height indexes (cm^2^m^−2^), where *n* = 24. The line of best fit is based on regression equation analysis, where *R*^2^ = 0.323. Graph **(B)** depicts a Bland–Altman plot and analysis of the averages between skeletal muscle cross-sectional area measured by CT at T4 and L3 as indexes of height (cm^2^m^−2^) against the differences between these measures (L3 minus T4), where *n* = 24. Mean difference ± standard deviation = −23.7 ± 5.2 cm^2^m^−2^. Graph **(C)** is an x–y scatter plot of the concordance between mean skeletal muscle attenuation (HU) measured by CT at T4 and L3, where *n* = 24. The line of best fit is shown, based on regression equation analysis, where *R*^2^ = 0.698. Graph **(D)** depicts a Bland–Altman plot and analysis of the averages between mean skeletal muscle attenuation (HU) measured by CT at T4 and L3 against the differences between these measures (L3 minus T4), where *n* = 24. Mean difference ± standard deviation = −3.14 ± 5.97 HU. In graphs **(B,D)**, no difference (blue interrupted line), mean difference (red line), and 95% upper and lower limits of agreement (mean difference ± 1.96*standard deviation, green lines) are indicated. Arrows indicate two overlapping data points.

The concordance between skeletal muscle indexes (skeletal muscle per CSA adjusted by height) measured by CT at T4 and L3 is shown in Figure 6A. Spearman’s rank correlation coefficient demonstrates a weakly positive correlation between skeletal muscle index measured from CSAs at T4 and L3 (R_s_ = 0.578, *p* = 0.003), suggesting that CT analysis at T4 and L3 ranks patients by skeletal muscle quantity less similarly than by TAT. The Bland–Altman analysis demonstrates that skeletal muscle index was 23.6 cm^2^m^−2^ higher using T4 compared to using L3 (see Figure 6B), indicating that increased muscle quantity was observed in the thoracic compared to the abdominal region. The concordance between muscle attenuation measured by CT at T4 and L3 is shown in Figure 6C. A strong positive correlation was shown between muscle attenuation measured by CT at T4 and L3 by Spearman’s rank correlation coefficient analysis (R_s_ = 0.840, *p* < 0.001), suggesting that muscle attenuation is similar between CT slices at T4 and L3. The Bland–Altman analysis (see Figure 6D) demonstrates that, on average, muscle attenuation is slightly lower, by 3.14 HU, in abdominal skeletal muscle at L3 compared to thoracic skeletal muscle at T4.

### 3.5 Summary of body composition parameters measured from the T4 and L3 cross-sectional areas

The comparison of the markers of body composition parameters by slice in this cohort is summarised in Table 4. Analysis using paired difference T-tests demonstrated significant differences between CSA measures at T4 and L3 as the TAT index (*p* < 0.001), VAT index (*p* < 0.001), skeletal muscle index (*p* < 0.001), and skeletal muscle attenuation (*p* = 0.017) from CT-derived transverse image CSAs. No statistical differences were observed between measures at T4 and L3 as subcutaneous and IMAT indexes.

**Table 4:** Body composition parameters of the cohort.

Body composition variable	Number of participants (*n*)	Mean ± standard deviation	Range	Paired difference *T*-test
Body mass index (kg/m^2^)	24	28.9 ± 5.4	19.5–41.8	
Total adipose tissue CSA at T4 (cm^2^)	19	248.0 ± 105.9	70.6–481.4	Mean difference = 129.6 (*p* < 0.001)
Total adipose tissue CSA at L3 (cm^2^)	19	377.6 ± 175.0	96.1–749.7	
Total adipose tissue index at T4 (cm^2^m^−2^)	19	90.1 ± 34.7	25.8–164.1	Mean difference = 47.0 (*p* < 0.001)
Total adipose tissue index at L3 (cm^2^m^−2^)	19	137.2 ± 58.8	35.1–248.0	
Subcutaneous adipose tissue CSA at T4 (cm^2^)	19	225.5 ± 97.0	61.6–436.0	Mean difference = 13.3 (*p* = 0.247)
Subcutaneous adipose tissue CSA at L3 (cm^2^)	19	238.7 ± 107.2	60.6–469.0	
Subcutaneous adipose tissue index at T4 (cm^2^m^−2^)	19	81.9 ± 32.0	22.5–148.6	Mean difference = 4.5 (*p* = 0.276)
Subcutaneous adipose tissue index at L3 (cm^2^m^−2^)	19	86.4 ± 35.6	22.1–155.1	
Intramuscular adipose tissue CSA at T4 (cm^2^)	19	17.6 ± 8.3	6.2–39.1	Mean difference = 0.8 (*p* = 0.789)
Intramuscular adipose tissue CSA at L3 (cm^2^)	19	18.4 ± 13.7	4.7–66.6	
Intramuscular adipose tissue index at T4 (cm^2^m^−2^)	19	6.5 ± 3.0	2.3–13.3	Mean difference = 0.2 (*p* = 0.824)
Intramuscular adipose tissue index at L3 (cm^2^m^−2^)	19	6.7 ± 4.8	1.7–23.6	
Visceral adipose tissue CSA at T4 (cm^2^)	19	4.9 ± 2.3	2.2–9.9	Mean difference = 115.6 (*p* < 0.001)
Visceral adipose tissue CSA at L3 (cm^2^)	19	120.5 ± 69.7	17.6–255.8	
Visceral adipose tissue index at T4 (cm^2^m^−2^)	19	1.8 ± 0.8	0.8–3.5	Mean difference = 42.3 (*p* < 0.001)
Visceral adipose tissue index at L3 (cm^2^m^−2^)	19	44.0 ± 24.3	6.1–87.2	
Skeletal muscle CSA at T4 (cm^2^)	24	169.6 ± 18.3	143.3–215.0	Mean difference = 63.7 (*p* < 0.001)
Skeletal muscle CSA at L3 (cm^2^)	24	105.9 ± 13.3	87.9–133.5	
Skeletal muscle index at T4 (cm^2^/m^2^)	24	62.8 ± 6.4	50.8–76.3	Mean difference = 23.7 (*p* < 0.001)
Skeletal muscle index at L3 (cm^2^/m^2^)	24	39.1 ± 3.9	32.8–46.8	
Mean skeletal muscle attenuation at T4 (HU)	24	39.07 ± 7.92	22.43–53.44	Mean difference = 3.14 (*p* = 0.017)
Mean skeletal muscle attenuation at L3 (HU)	24	35.93 ± 10.70	20.70–53.93	

This table summarises the body composition parameters of the study cohort by BMI and CT analysis at T4 and L3.

## 4 Discussion

This study is the first to compare body composition from CT slices at T4 and L3 in a breast cancer cohort, and our data demonstrate that transverse CT slices at T4 and L3 are highly concordant measures of TAT quantity.

In this cohort of patients with early breast cancer, total and VAT per CSA was significantly higher when measured at L3 than T4, reflecting increased visceral adipose deposition in the abdominal region compared to the thorax. High concordance was found between total, intramuscular, and SAT per CSA measured in the thorax (T4) and the abdomen (L3), while lower concordance was observed with visceral adiposity. This aspect suggests that these thoracic and abdominal CT slices are more comparable when ranking total, intramuscular, and subcutaneous than visceral adiposity. This may be partially explained by the observation that visceral adiposity increased; a greater difference was observed between visceral adiposity at T4 and L3, suggesting underlying differences in visceral adipose deposition patterns in the thorax and the abdomen. Although TAT is a cumulative measure of intramuscular, subcutaneous, and visceral adiposity, the concordance between total adiposity and visceral adiposity was high, despite the lower concordance observed in visceral adiposity between T4 and L3 in this cohort. In women with haematological malignancies who underwent allogeneic haemopoietic stem cell transplants, Mishra et al. (26) found a weaker correlation between total and SAT measured at T4 and L3 (*r*^2^ = 0.79, *p* < 0.001 and *r*^2^ = 0.69, *p* < 0.001, respectively). However, Mishra et al. excluded intramuscular and VAT at T4 or IMAT at L3 in their calculation of “total adipose tissue”, thereby limiting comparison between findings.

Following the adjustment for height, more skeletal muscles were observed at T4 than at L3. This aspect reflects well-established differences in muscular anatomy in the thorax compared to the abdominal region. As shown in Figure 2, the abdominal slice (L3) consists primarily of postural axial paraspinal and structural abdominal wall skeletal muscle groups with smaller CSAs, compared to the larger appendicular shoulder girdle muscles, and chest wall and paraspinal muscles comprising the thoracic slice (T4). Higher muscularity at T4 than at L3 was similarly observed by Mishra et al. and also found in a mixed-gender cohort of patients undergoing liver metastasis resection for colorectal cancer and in women with advanced small cell lung cancer (25, 26, 34). A poor degree of concordance was observed in this cohort between skeletal muscle following adjustment for height when measured in the thorax (T4) and the abdomen (L3). The anatomical differences in musculature between the thorax and the abdomen may contribute to the poorer concordance between the ranking of patients’ skeletal muscles using T4 and L3 slices. Mishra et al. observed a lower correlation for skeletal muscle quantity in women with haematological malignancies (*r*^2^ = 0.33, *p* < 0.001), while Grøberg et al. reported that the skeletal muscle index at T4 was only weakly predictive of skeletal muscle index at L3 in women with advanced non-small cell lung cancer (*r*^2^ = 0.28) (26, 34). However, direct comparison with the studies by Mishra et al. was constrained as skeletal muscle quantity was not adjusted by height, and Spearman’s rank correlation analysis was not conducted.

Conversely, Van der Kroft et al. found a higher correlation (*r* = 0.78, *p* < 0.001) between skeletal muscle index measured at T4 and L3; however, in the study by Van der Kroft et al. (25), Spearman’s rank correlation analysis was not conducted, and the study cohort consisted of a mixed population of men and women with colorectal cancer undergoing elective liver metastasis resection. On average, muscle attenuation was slightly lower at L3, but reasonably strong concordance was observed in our cohort between skeletal muscle attenuation at T4 and L3, suggesting that muscle attenuation is comparable between muscles in the thorax and abdomen. This fact may indicate that factors determining muscle attenuation, including intramuscular fat infiltration, affect muscle groups similarly throughout the body. The same studies by Van der Kroft et al. and Mishra et al. found a weaker correlation between skeletal muscle attenuation at T4 and L3 (*r* = 0.43, *p* < 0.001 and *r*^2^ = 0.58, *p* < 0.001 respectively), while Grøberg et al. found muscle attenuation at T4 weakly predicted muscle attenuation at L3 (*r*^2^ = 0.58) (25, 26, 34). All three studies support the result from this study, with average muscle attenuation being higher at T4 compared to L3. These findings suggest that attenuation in muscle groups in the thorax may vary in muscle groups in the abdomen, but further research is needed to compare individual muscle groups.

The patient demographics of this cohort of women are comparable. In terms of age for the national breast cancer population in the United Kingdom, majority of breast cancer diagnoses in 2017 were in women aged over 50 years, with a median age of 62 years at diagnosis (35). In this cohort, 41.7% of women underwent breast-conserving surgery, compared to 57% of women who underwent surgery nationally (35). The tumour characteristics of this cohort were highly representative in terms of ER and HER2 status, with national UK breast population figures of women who undergo surgical treatment at 84 and 15%, respectively (35). However, this cohort had more higher grade tumours and higher rates of lymph node involvement compared to national figures of women who underwent surgical treatment, suggesting that this cohort has the more advanced local regional disease (35). Manual analysis of CT images conducted by researchers who have received training in anatomy facilitates the identification and exclusion of small tissue structures such as lymphatics within musculature or fat from radiological images for deriving the estimates of body composition measurements. However, this methodology remains susceptible to misidentification and human error. We attempted to minimise the impact of human error and researcher variability by having two independent researchers and agreeing on protocols. The variability between some measurements, as demonstrated in Figures 3, 4, highlights this importance. Recent studies in body composition have aimed to automate CT analysis using computer algorithms, which may be more time and resource-efficient and eliminate inter-researcher variability but may incorrectly include these smaller structures, leading to measurement inaccuracies (36–42). A limitation of this study is that patients were only included if they underwent routine chest–abdomen–pelvis CT scans, which is not recommended according to UK guidelines unless metastatic disease is suspected (21, 22). This resulted in a limited sample size of 24 patients. As only patients who were considered at higher risk of having metastatic disease receive CT scans, there is likely a bias whereby the study cohort consists of patients with more advanced local, regional disease compared to the general breast cancer population, which is consistent with the patterns of tumour grade and lymph node involvement observed.

This study is a single-centre study, so it is not possible to generalise the results to other specific populations. A further area for improvement of this study is that some CT images at T4 were unilaterally incomplete where tissue exceeded the field of view, necessitating that only the complete, contralateral half image was analysed, and results were doubled accordingly. Though this methodology has been utilised in other published studies, there needs to be more published evidence that muscle and adipose tissue are symmetrically distributed in the trunk. Therefore, this method may lead to inaccuracies (34, 43, 44).

## 5 Conclusion

These results demonstrate that in our cohort of patients with early breast cancer, CT analysis at T4 and L3 ranks patients with breast cancer similarly by TAT quantity and muscle attenuation per CSA but is less concordant for visceral adiposity and skeletal muscle quantity. Body composition markers measured from transverse CT slices at T4 are potentially of greater availability in patients with breast cancer, as routine thoracic CT scans are performed more frequently in this patient group for radiotherapy planning. However, further research is needed to determine whether body composition metrics analysed from transverse CT images at T4 are similarly predictive for clinical outcomes in patients with breast cancer before clinical translation.

## Data availability statement

The raw data supporting the conclusions of this article will be made available by the authors, without undue reservation.

## Ethics statement

The studies involving humans were approved by REC reference number: 14/EE/1297. The studies were conducted in accordance with the local legislation and institutional requirements. The participants provided their written informed consent to participate in this study.

## Author contributions

AD: Writing – review & editing, Methodology, Formal analysis, Data curation. LN: Writing – original draft, Validation, Formal analysis. AT: Writing – review & editing, Validation, Formal analysis, Data curation. AM: Writing – review & editing, Validation, Methodology. CS: Writing – review & editing, Methodology, Data curation. JL: Writing – review & editing, Methodology, Data curation. JA: Writing – review & editing, Methodology, Data curation. KD: Writing – review & editing, Project administration, Investigation, Data curation. DL: Writing – review & editing, Methodology, Conceptualization. AH: Writing – review & editing, Investigation, Data curation. SW: Writing – review & editing, Visualization, Validation, Supervision, Methodology, Conceptualization. EC: Writing – original draft, Visualization, Supervision, Methodology, Conceptualization. RC: Writing – original draft, Visualization, Supervision, Methodology, Funding acquisition, Conceptualization.

## References

[1] Lee K Kruper L Dieli-Conwright CM Mortimer JE. The impact of obesity on breast Cancer diagnosis and treatment. Curr Oncol Rep. (2019) 21:41. doi: 10.1007/s11912-019-0787-1 30919143 PMC6437123

[2] Protani M Coory M Martin JH. Effect of obesity on survival of women with breast cancer: systematic review and meta-analysis. Breast Cancer Res Treat. (2010) 123:627–35. doi: 10.1007/s10549-010-0990-0, PMID: 20571870

[3] Ewertz M Jensen MB Gunnarsdóttir KÁ Højris I Jakobsen EH Nielsen D. Effect of obesity on prognosis after early-stage breast Cancer. JCO. (2010) 29:25–31. doi: 10.1200/JCO.2010.29.7614 21115856

[4] Copson ER Cutress RI Maishman T Eccles BK Gerty S Stanton L. Obesity and the outcome of young breast cancer patients in the UK: the POSH study. Ann Oncol. (2015) 26:101–12. doi: 10.1093/annonc/mdu509 25361993

[5] Garland M Hsu FC Clark C Chiba A Howard-McNatt M. The impact of obesity on outcomes for patients undergoing mastectomy using the ACS-NSQIP data set. Breast Cancer Res Treat. (2018) 168:723–6. doi: 10.1007/s10549-017-4651-4, PMID: 29327298

[6] Renehan AG Harvie M Cutress RI Leitzmann M Pischon T Howell S. How to manage the obese patient with Cancer. J Clin Oncol. (2016) 34:4284–94. doi: 10.1200/JCO.2016.69.1899 27903151

[7] Invernizzi M Lippi L Folli A Turco A Zattoni L Maconi A. Integrating molecular biomarkers in breast cancer rehabilitation. What is the current evidence? A systematic review of randomized controlled trials. Front Mol Biosci. (2022) 9:930361. doi: 10.3389/fmolb.2022.930361, PMID: 36158576 PMC9493088

[8] Kerlikowske K Walker R Miglioretti DL Desai A Ballard-Barbash R Buist DSM. Obesity, mammography use and accuracy, and advanced breast cancer risk. J Natl Cancer Inst. (2008) 100:1724–33. doi: 10.1093/jnci/djn388, PMID: 19033562 PMC2734114

[9] Blair CK Wiggins CL Nibbe AM Storlie CB Prossnitz ER Royce M. Obesity and survival among a cohort of breast cancer patients is partially mediated by tumor characteristics. Npj. Breast Cancer. (2019) 5:1–7. doi: 10.1038/s41523-019-0128-4 31602394 PMC6775111

[10] Budman DR Berry DA Cirrincione CT Craig HI Wood WC Weiss RB. Dose and dose intensity as determinants of outcome in the adjuvant treatment of breast Cancer. JNCI J Natl Cancer Inst. (1998) 90:1205–11. doi: 10.1093/jnci/90.16.1205 9719081

[11] Griggs JJ Sorbero MES Lyman GH. Undertreatment of obese women receiving breast Cancer chemotherapy. Arch Intern Med. (2005) 165:1267–73. doi: 10.1001/archinte.165.11.1267, PMID: 15956006

[12] Roberto M Barchiesi G Resuli B Verrico M Speranza I Cristofani L. Sarcopenia in breast Cancer patients: a systematic review and Meta-analysis. Cancers (Basel). (2024) 16:596. doi: 10.3390/cancers16030596, PMID: 38339347 PMC10854936

[13] Mallard J Hucteau E Hureau TJ Pagano AF. Skeletal muscle deconditioning in breast Cancer patients undergoing chemotherapy: current knowledge and insights from other cancers. Front Cell Dev Biol. (2021) 9:719643. doi: 10.3389/fcell.2021.719643, PMID: 34595171 PMC8476809

[14] Heetun A Cutress RI Copson ER. Early breast cancer: why does obesity affect prognosis? Proc Nutr Soc. (2018) 77:369–81. doi: 10.1017/S0029665118000447, PMID: 29860965

[15] James FR Wootton S Jackson A Wiseman M Copson ER Cutress RI. Obesity in breast cancer – what is the risk factor? Eur J Cancer. (2015) 51:705–20. doi: 10.1016/j.ejca.2015.01.057 25747851

[16] Caan BJ Cespedes Feliciano EM Prado CM Alexeeff S Kroenke CH Bradshaw P. Association of Muscle and Adiposity Measured by computed tomography with survival in patients with nonmetastatic breast Cancer. JAMA Oncol. (2018) 4:798–804. doi: 10.1001/jamaoncol.2018.0137, PMID: 29621380 PMC6584322

[17] Deluche E Leobon S Desport JC Venat-Bouvet L Usseglio J Tubiana-Mathieu N. Impact of body composition on outcome in patients with early breast cancer. Support Care Cancer. (2018) 26:861–8. doi: 10.1007/s00520-017-3902-6, PMID: 28948392 PMC5785600

[18] Peng YC Wu CH Tien YW Lu TP Wang YH Chen BB. Preoperative sarcopenia is associated with poor overall survival in pancreatic cancer patients following pancreaticoduodenectomy. Eur Radiol. (2021) 31:2472–81. doi: 10.1007/s00330-020-07294-7, PMID: 32974690

[19] Ying P Jin W Wu X Cai W. Association between CT-quantified body composition and recurrence, survival in nonmetastasis colorectal Cancer patients underwent regular chemotherapy after surgery. Biomed Res Int. (2021) 2021:1–8. doi: 10.1155/2021/6657566 33834071 PMC8016588

[20] Cespedes Feliciano EM Avrutin E Caan BJ Boroian A Mourtzakis M. Screening for low muscularity in colorectal cancer patients: a valid, clinic-friendly approach that predicts mortality. J Cachexia Sarcopenia Muscle. (2018) 9:898–908. doi: 10.1002/jcsm.12317, PMID: 30066490 PMC6204585

[21] National Institute for Health and Care Excellence. Recommendations-advanced breast cancer: diagnosis and treatment, guidance, NICE [internet]. NICE; 2009 [cited 2021 Dec 23]. Available at: https://www.nice.org.uk/guidance/cg81/chapter/recommendations

[22] National Institute for Health and Care Excellence. Overview- early and locally advanced breast cancer: diagnosis and management, guidance, NICE [internet]. NICE; [cited 2021 Dec 28]. Available at: https://www.nice.org.uk/guidance/ng101

[23] Chand N Cutress RI Oeppen RS Agrawal A. Staging investigations in breast Cancer: collective opinion of UK breast surgeons. Int J Breast Cancer. (2013) 2013:506172:1–8. doi: 10.1155/2013/506172 24349790 PMC3853040

[24] Cancer Research UK. (2015) [cited 2021 Dec 4]. Breast cancer treatment statistics. Available at: https://www.cancerresearchuk.org/health-professional/cancer-statistics/statistics-by-cancer-type/breast-cancer/diagnosis-and-treatment

[25] Van der Kroft G van Dijk DPJ Rensen SS Van Tiel FH de Greef B West M. Low thoracic muscle radiation attenuation is associated with postoperative pneumonia following partial hepatectomy for colorectal metastasis. HPB (Oxford). (2020) 22:1011–9. doi: 10.1016/j.hpb.2019.10.1532, PMID: 31735648

[26] Mishra A Bigam KD Extermann M Faramand R Thomas K Pidala JA. Sarcopenia and low muscle radiodensity associate with impaired FEV1 in allogeneic haematopoietic stem cell transplant recipients. J Cachexia Sarcopenia Muscle. (2020) 11:1570–9. doi: 10.1002/jcsm.12604, PMID: 32729255 PMC7749567

[27] Huang WJ Zhang ML Wang W Jia QC Yuan JR Zhang X. Preoperative pectoralis muscle index predicts distant metastasis-free survival in breast Cancer patients. Front Oncol. (2022) 12:854137. doi: 10.3389/fonc.2022.854137 35574329 PMC9098931

[28] Common terminology criteria for adverse events (CTCAE) | protocol development | CTEP [internet]. [cited 2021 Dec 11]. Available at: https://ctep.cancer.gov/protocoldevelopment/electronic_applications/ctc.htm

[29] Netter F. Vertebral column Altas of human anatomy. Sixth ed. Philadelphia: Elsevier Saunders; (2014). 153–157.

[30] Alberta protocol: user’s manual v5.0. Magog, Quebec, Canada: TomoVision (2017).

[31] Heymsfield SB Gallagher D Mayer L Beetsch J Pietrobelli A. Scaling of human body composition to stature: new insights into body mass index. Am J Clin Nutr. (2007) 86:82–91. doi: 10.1093/ajcn/86.1.82, PMID: 17616766 PMC2729090

[32] Heymsfield SB Heo M Thomas D Pietrobelli A. Scaling of body composition to height: relevance to height-normalized indexes. Am J Clin Nutr. (2011) 93:736–40. doi: 10.3945/ajcn.110.007161, PMID: 21248190

[33] Body mass index—BMI [internet]. [cited 2021 Dec 8]. Available at: https://www.euro.who.int/en/health-topics/disease-prevention/nutrition/a-healthy-lifestyle/body-mass-index-bmi

[34] Grønberg BH Sjøblom B Wentzel-Larsen T Baracos VE Hjermstad MJ Aass N. A comparison of CT based measures of skeletal muscle mass and density from the Th4 and L3 levels in patients with advanced non-small-cell lung cancer. Eur J Clin Nutr. (2019) 73:1069–76. doi: 10.1038/s41430-018-0325-5 30254241

[35] Lawrence G Kearins O Lagord C Cheung S Sidhu J Sagar C. The second all breast cancer report-focusing on inequalities: variation in breast cancer outcomes with age and deprivation. National Cancer Intelligence Network; (2011) [cited 2021 Dec 28]. Available at: http://www.ncin.org.uk/search/second+all+breast

[36] Dabiri S Popuri K Cespedes Feliciano EM Caan BJ Baracos VE Beg MF. Muscle segmentation in axial computed tomography (CT) images at the lumbar (L3) and thoracic (T4) levels for body composition analysis. Comput Med Imaging Graph. (2019) 75:47–55. doi: 10.1016/j.compmedimag.2019.04.007, PMID: 31132616 PMC6620151

[37] Weston AD Korfiatis P Kline TL Philbrick KA Kostandy P Sakinis T. Automated abdominal segmentation of CT scans for body composition analysis using deep learning. Radiology. (2019) 290:669–79. doi: 10.1148/radiol.2018181432, PMID: 30526356

[38] Koitka S Kroll L Malamutmann E Oezcelik A Nensa F. Fully automated body composition analysis in routine CT imaging using 3D semantic segmentation convolutional neural networks. Eur Radiol. (2021) 31:1795–1804. doi: 10.1007/s00330-020-07147 32945971 PMC7979624

[39] Nowak S Faron A Luetkens JA Geißler HL Praktiknjo M Block W. Fully automated segmentation of connective tissue compartments for CT-based body composition analysis: a deep learning approach. Investig Radiol. (2020) 55:357–66. doi: 10.1097/RLI.0000000000000647, PMID: 32369318

[40] Dabiri S Popuri K Ma C Chow V Feliciano EMC Caan BJ. Deep learning method for localization and segmentation of abdominal CT. Comput Med Imaging Graph. (2020) 85:101776. doi: 10.1016/j.compmedimag.2020.101776, PMID: 32862015 PMC7803471

[41] Blanc-Durand P Campedel L Mule S Jegou S Luciani A Pigneur F. Prognostic value of anthropometric measures extracted from whole-body CT using deep learning in patients with non-small-cell lung cancer. Eur Radiol. (2020) 30:3528–37. doi: 10.1007/s00330-019-06630-w 32055950

[42] Zopfs D Bousabarah K Lennartz S Santos DPD Schlaak M Theurich S. Evaluating body composition by combining quantitative spectral detector computed tomography and deep learning-based image segmentation. Eur J Radiol. (2020) 130:109153. doi: 10.1016/j.ejrad.2020.109153, PMID: 32717577

[43] Buckner SL Abe T Counts BR Dankel SJ Barnett BE Loenneke JP. Muscle and fat mapping of the trunk: a case study. J Ultrasound. (2015) 18:399–405. doi: 10.1007/s40477-015-0179-9, PMID: 26550077 PMC4630266

[44] Kullberg J Brandberg J Angelhed JE Frimmel H Bergelin E Strid L. Whole-body adipose tissue analysis: comparison of MRI, CT and dual energy X-ray absorptiometry. BJR. (2009) 82:123–30. doi: 10.1259/bjr/80083156, PMID: 19168691

