# Supplementary material for: Assessment of body composition in breast cancer patients: concordance between transverse computed tomography analysis at the fourth thoracic and third lumbar vertebrae
Source: Front Nutr. 2024 Apr 23;11:1366768. doi: 10.3389/fnut.2024.1366768 (PMC11074467; doi:10.3389/fnut.2024.1366768)
Supplement: Supplementary file 1 [file Data_Sheet_1.docx]

**Supplementary material 1)** Protocol for Selecting an L3 slice through Radiant™ DICOM Viewer software


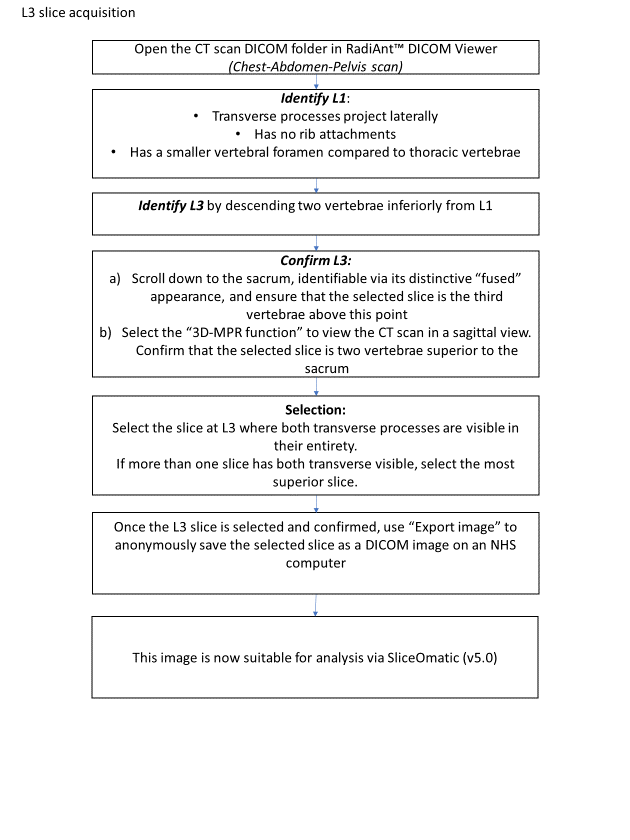


**Supplementary material 2)** Protocol for the Body Composition Analysis of CT scans at L3 using Sliceomatic™ (v5.0) software to measure SAT, VAT, IMAT, SM quantity and attenuation.


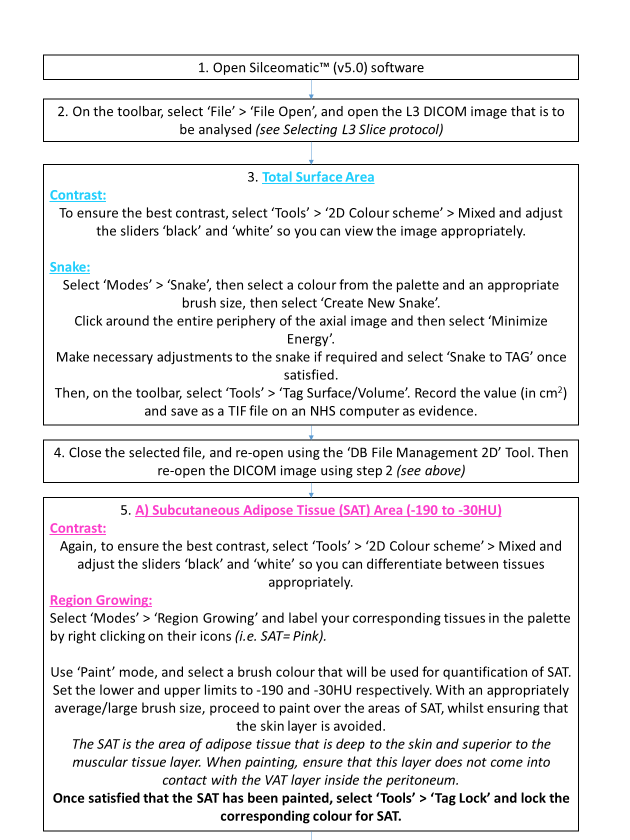


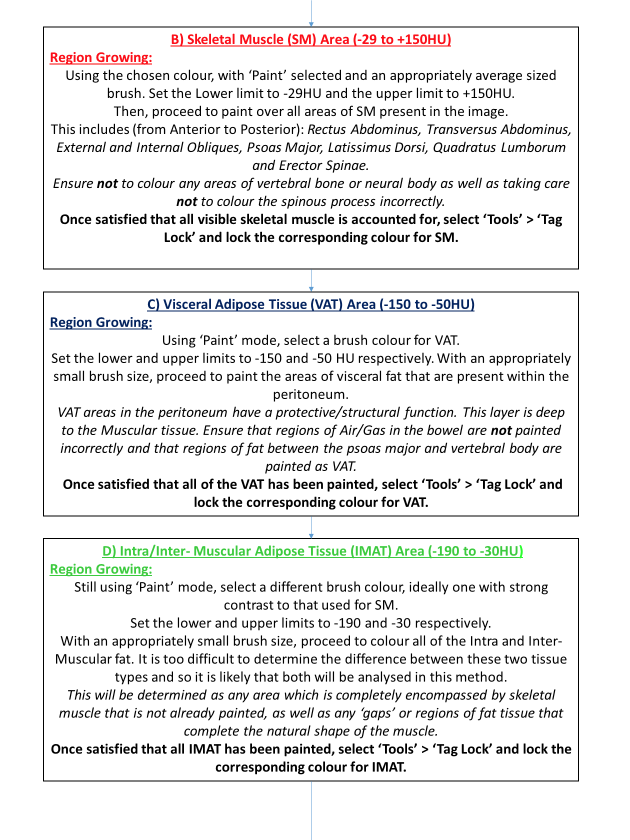


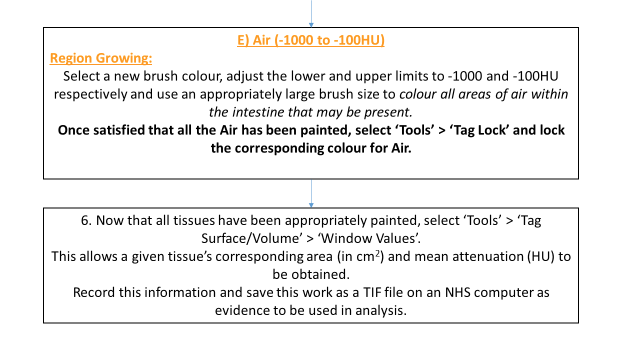


**Supplementary material 3)** Protocol for the selection of a T4 CT image using Radiant™ DICOM Viewer software


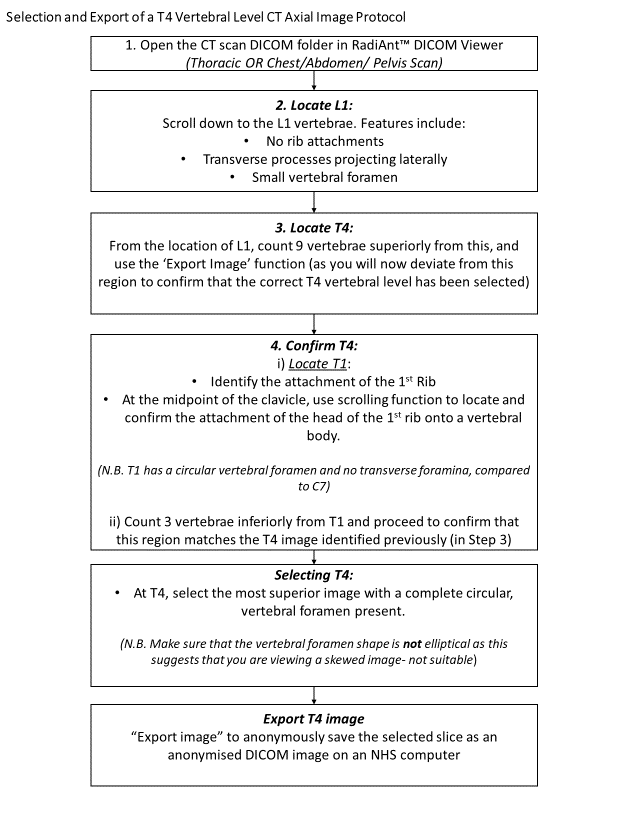


**Supplementary material 5)** Protocol for the Body Composition Analysis of CT scans at T4 using Sliceomatic™ (v5.0) software to measure SAT, VAT, IMAT, SM quantity and attenuations.


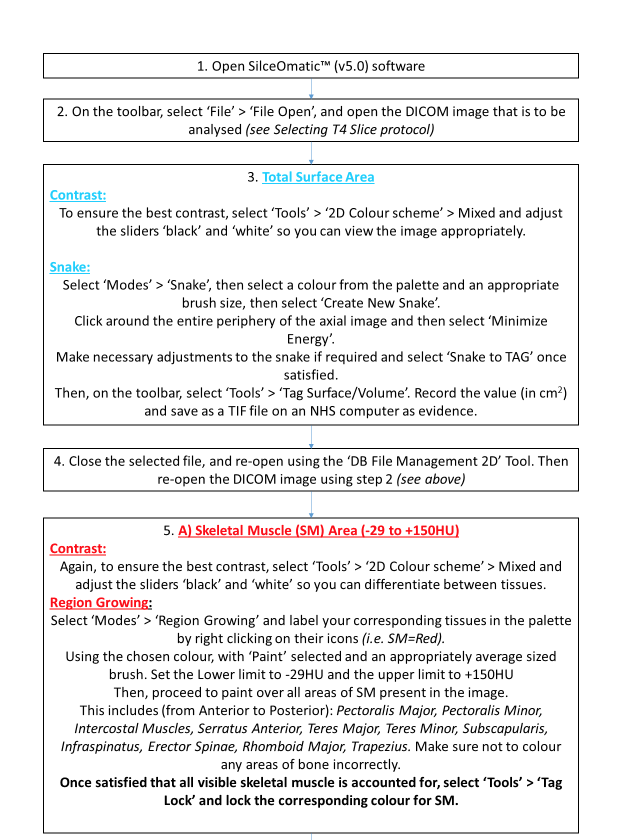


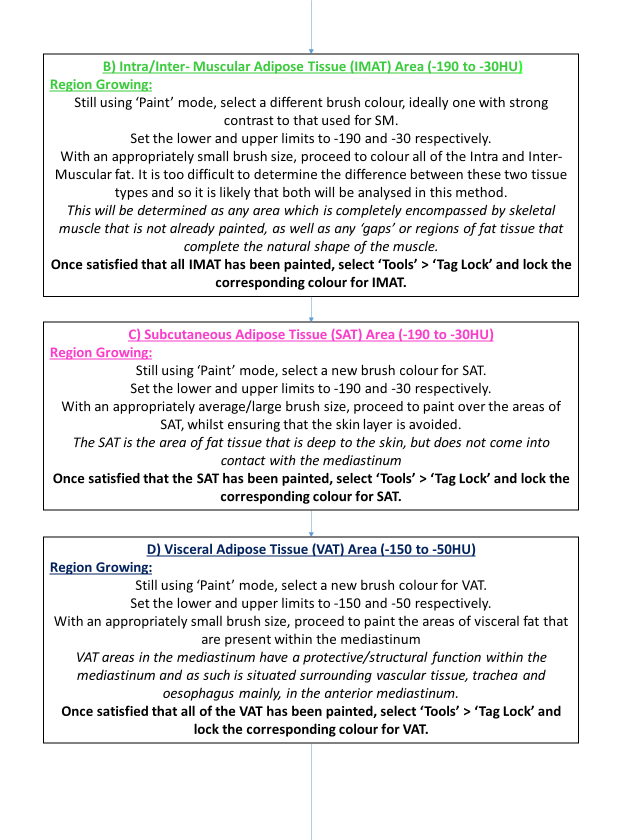


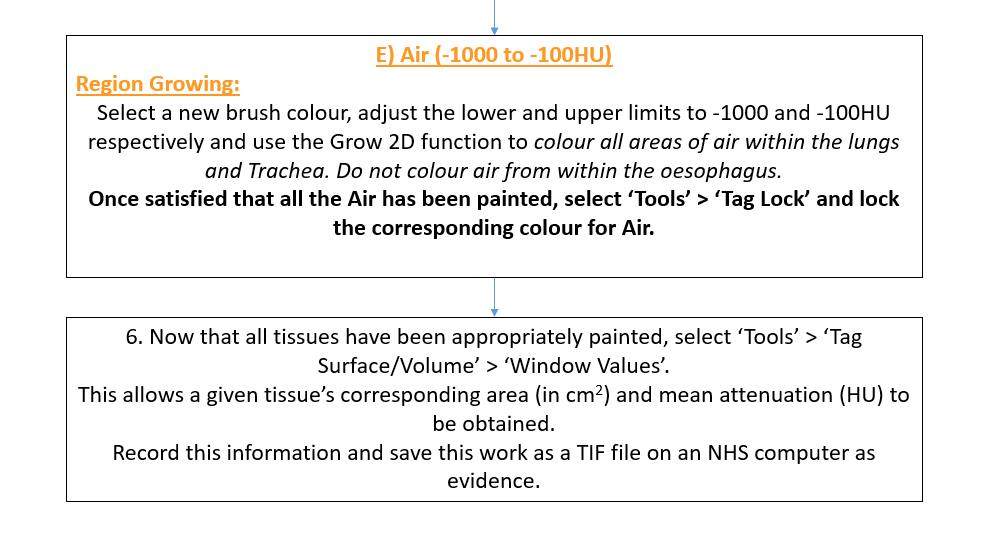


**Supplementary material 6)** Protocol for CT scan analysis under circumstances where only a half-image has been available


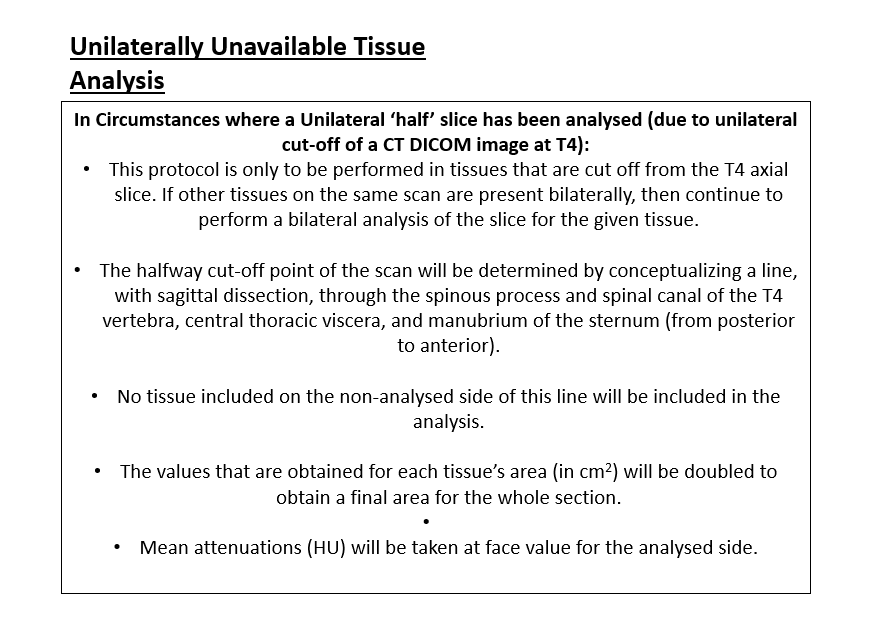


**Supplementary material 7)** Equations used to calculate skeletal muscle and adipose tissue index values.

1. $Skeletal muscle \left( SM \right) index=SMCSAi \left( {cm}^{2}m^{-2} \right)= \frac{SMCSA({cm}^{2})}{{height}^{2}{(m}^{2})}$
2. $Total adipose tissue (TAT) index=TATCSAi \left( {cm}^{2}m^{-2} \right)= \frac{TATCSA({cm}^{2})}{{height}^{2}{(m}^{2})}$
3. $Visceral adipose tissue (VAT) index=VATCSAi \left( {cm}^{2}m^{-2} \right)= \frac{VATCSA({cm}^{2})}{{height}^{2}{(m}^{2})}$
4. $Subcutaneous adipose tissue \left( SAT \right) index=SATCSAi \left( {cm}^{2}m^{-2} \right)= \frac{SATCSA\left( {cm}^{2} \right)}{{height}^{2}{(m}^{2})}$
5. $Intramuscular adipose tissue (IMAT) index=IMATCSAi \left( {cm}^{2}m^{-2} \right)=\frac{IMATCSA({cm}^{2})}{{height}^{2}{(m}^{2})}$
